# Mechanical force system of double key loop with finite element analysis

**DOI:** 10.1186/s12903-021-01657-2

**Published:** 2021-06-13

**Authors:** Jiali Liu, Duanqiang Zhang, Linyu Xu, Senxin Cai, Jinquan Guo, Jiang Chen, Jiehua Su

**Affiliations:** 1 Fujian Key Laboratory of Oral Diseases & Fujian Provincial Engineering Research Center of Oral Biomaterial & Stomatological Key Lab of Fujian College and University, School and Hospital of Stomatology, Fujian Medical University, Fuzhou, China; 2 Institute of Stomatology & Research Center of Dental Esthetics and Biomechanics & Department of Orthodontics, School and Hospital of Stomatology, Gulou District, Fujian Medical University, No. 246 Yangqiaozhong Road, Fuzhou, 350002 China; 3 School of Mechanical Engineering and Automation, Fuzhou University, Fuzhou, China

**Keywords:** Finite element analysis, Double key loop, M/F ratio, Loop mechanics, Orthodontics

## Abstract

**Background:**

The mechanics of double key loop (DKL) are not well defined, and this finite element study was designed to explore its force system.

**Methods:**

A simplified 3-dimensional finite element model of single and double key loops with an archwire between the lateral incisor and second premolar was established in Ansys Workbench 17.0. Activation in Type-1 (retraction at the distal end), Type-2 (retraction at the distal key) and Type-3 (Type-2 plus ligation between keys) was simulated. The vertical force, load/deflection ratio and moment/force ratio of stainless-steel and titanium-molybdenum alloy (TMA) loops were calculated and compared.

**Results:**

The double key loop generated approximately 40% of the force of a single key loop. Type-2 loading of DKL showed a higher L/D ratio than Type-1 loading with a similar M/F ratio. Type-3 loading of DKL showed the highest M/F ratio with a similar L/D ratio as single key loop. The M/F ratio in Type-3 loading increased with the decreasing of retraction force. The DKL of TMA produced approximately 40% of the force and moment compared with those of SS in all loading types. When activated at equal distances below 1 mm, the M/F ratios of SS and TMA DKL with equal preactivation angles were almost the same.

**Conclusion:**

The M/F ratio on anterior teeth increases with the preactivation angle and deactivation of DKL. The M/F ratio at a certain distance of activation mainly depends on the preactivation angle instead of the wire material. TMA is recommended as a substitute for SS in DKL for a lower magnitude of force.

## Background

In orthodontic treatment with premolar extraction, sliding and closing loops are two major techniques to close the space. Although sliding mechanics is widely used in the clinic with the advantages of simplified mechanics, increased patient comfort and reduced chair time [1, 2], loop mechanics is still thought to be more efficient in controlling tooth movement patterns [3]. Orthodontic loops are frictionless, and all the generated force will be fully expressed against the brackets and finally to teeth after deactivation [4]. Some clinicians still prefer loop mechanics to sliding mechanics [5, 6].

Closing loops with different configurations such as T loops, teardrop loops, L loops, and mushroom loops are used in the clinic, and they need individual adjustments according to the clinical experience of orthodontists [4, 7]. The load deflection ratio (L/D), vertical force and moment-to-force ratio (M/F) are three important indexes for the evaluation of different loops, and the M/F ratio is the most critical index in loop mechanics. Generally, a force system with an M/F ratio of 7 mm induced controlled tipping, and an M/F ratio above 10 was required for translation of teeth [8, 9]. As reported in previous experimental and analytical studies, the M/F ratio varied with the wire material, cross section, height, width and configuration of loops [10–14]. Preactivation methods such as gable bends and vertical steps diversified the M/F ratio [15–18]. The M/F ratio was also reported to change with the distance of activation [19, 20].

Double key loop (DKL) is a special method advocated by John Parker to close space with straight wire appliance [21]. Normally, DKL is composed of two key holes, vertical loops at the mesial and distal interproximal positions of canines. Loading at the distal end, distal key and additional ligation between keys are the three major loading types of DKL, which provide advantages of flexible force system and effective control of anterior torque [22]. Retrospective clinical studies by Dr. Kim and Chen reported the high efficiency of DKL in the vertical and torque control of upper anterior teeth [23, 24]. Clinicians are interested in the mechanical properties of DKL, and several relevant studies have tried to explore precise control methods for DKL. Dobranszki used photoelastic models to compare the force response of teeth subjected to DKL and confirmed that vertical force on anterior teeth varied with the loading types [25]. Tábitha used finite element method to investigate the force and deformation of DKL, but no detailed M/F ratio results were provided [26].

To provide a preliminary guide for the application of DKL, finite element method was used in this study to explore the effect of wire material, loading types and preactivation angle on the mechanical force system of DKL.

## Methods

**Fig. 1:**
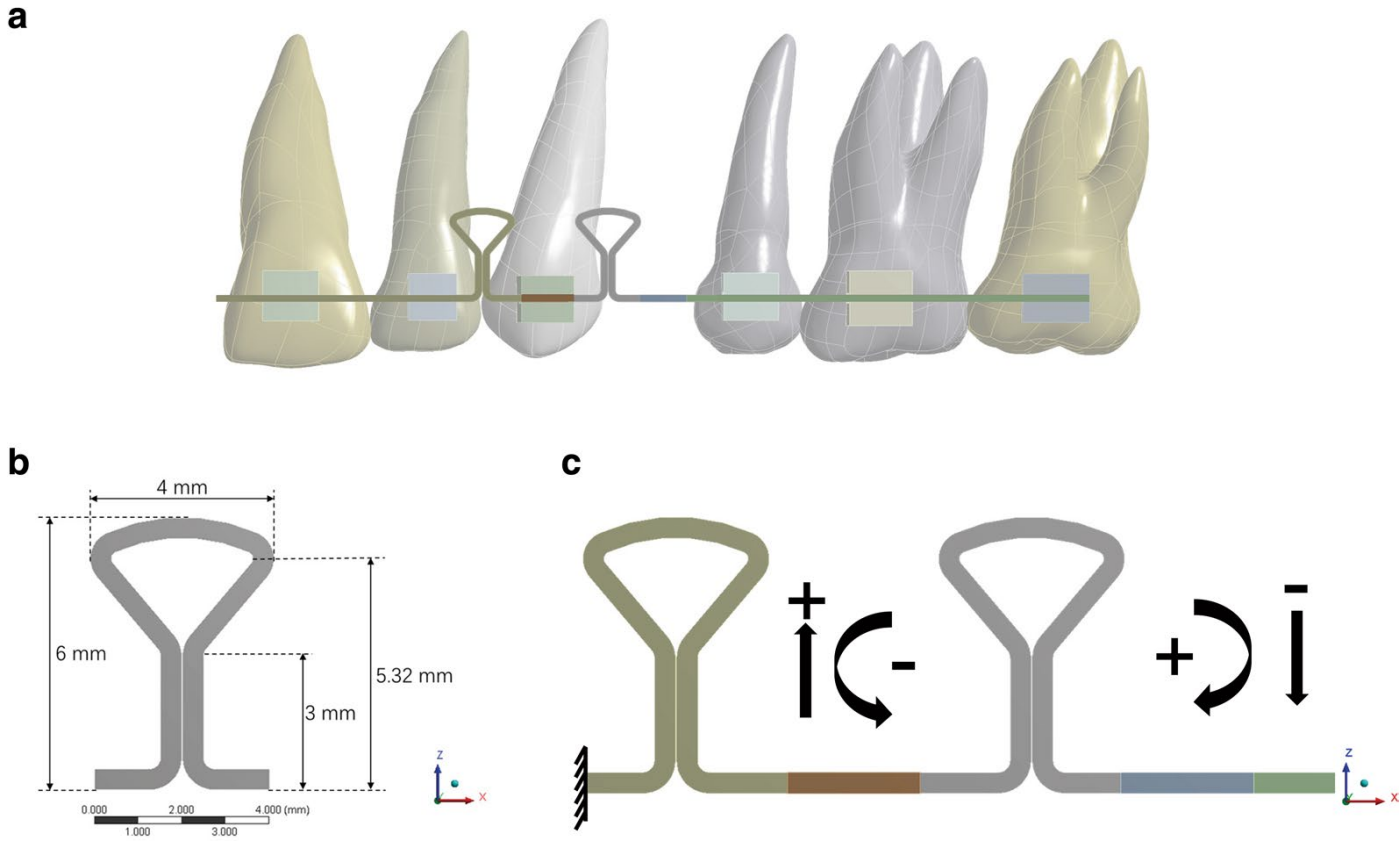
Configuration and dimensions of the double key loop (DKL): **a** Clinical use of DKL for closure of the upper premolar extraction space. **b** Dimension of the key loop, with a height of 6 mm and width of 4 mm. **c** Definition of forces and moments. Positive force indicated intrusion of teeth, and negative force indicated extrusion of teeth. Positive moments rotated the anterior tooth clockwise, and negative moments rotated the anterior tooth counterclockwise

**Fig. 2:**
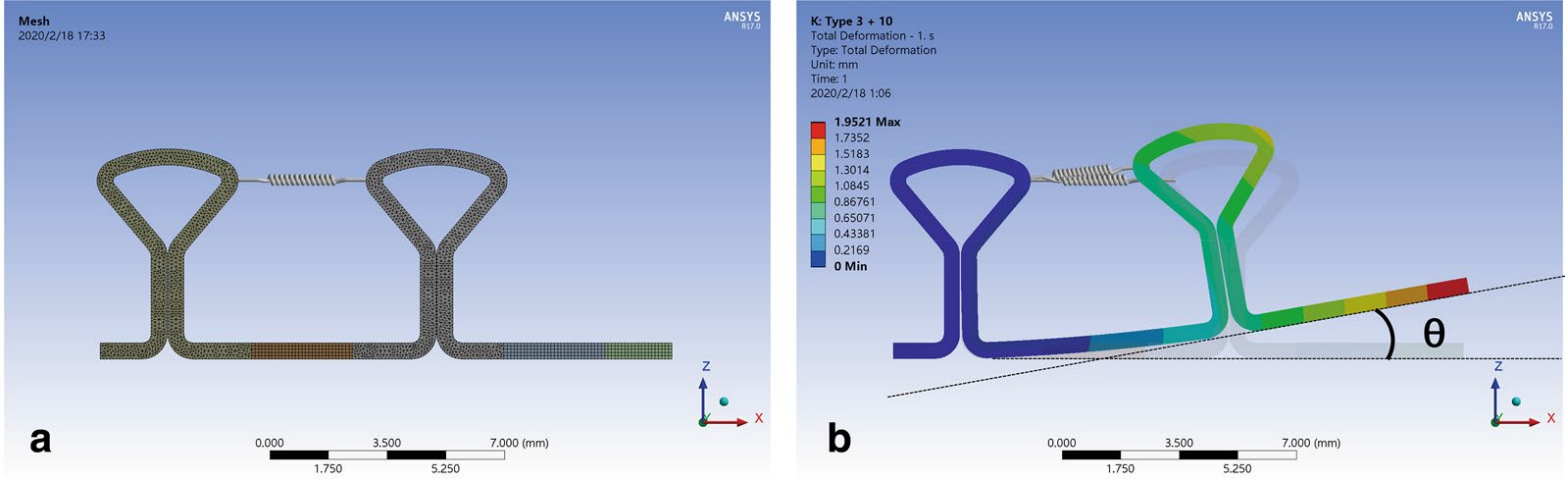
Meshing and preactivation of DKL. **a** Meshing of DKL model with refined elements. **b** Archwire with key loops was fixed on the mesial end, and the distal end was free. Simulative preactivation of DKL with a spring generated curvature between the mesial and distal archwires. The angle between the distal archwire and horizontal line was named as the preactivation angle (θ)

Archwire between the upper lateral incisor and second premolar with a single key loop and a double key loop was established in finite element analysis software Ansys Workbench 17.0 (ANSYS, USA). The cross-section of the archwire was 0.019 × 0.025 inch rectangular, and the configuration of the key loop is shown in Fig. 1. The height and width of the key loop were 6 mm and 4 mm, respectively. Key loops were at the mesial and distal interproximal positions of the canine. The archwire was divided into several parts, and bonded contacts were added between the parts to calculate the force and moments at specific positions. Automatic meshing was finished in Workbench, and 174,830 nodes and 108,039 elements were attained (Fig. 2a). Wire material was defined as stainless steel (SS) with an elastic property of Yang’s modulus 168 GPa, Poisson’s ratio 0.3, and titanium-molybdenum alloy (TMA) with Yang’s modulus 66 GPa, Poisson’s ratio 0.3[5, 27].

**Fig. 3:**
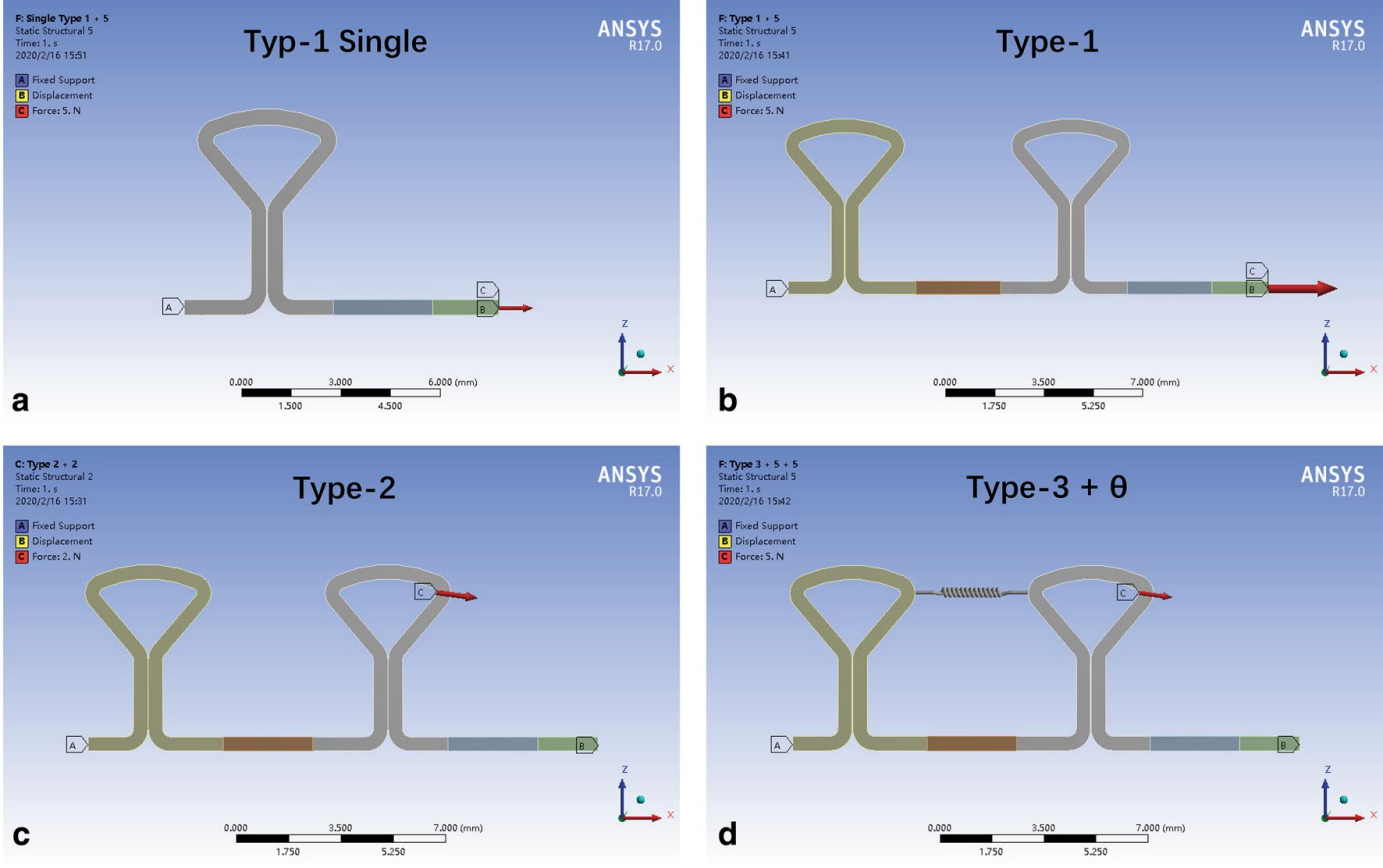
Loading conditions of key loops. Archwire with key loops was fixed on the mesial end, and the distal end was constrained in the vertical component. **a** Single key loop subjected to horizontal force at the distal end. **b** DKL subjected to horizontal force at the distal end. **c** DKL subjected to retraction force from the distal key to the tube of the second molar. **d** DKL subjected to retraction force from the distal key to the tube of the second molar plus a spring between key loops generating a preactivation angle (θ)

To simulate the activation of key loops, the mesial end was fixed in six degrees of freedom (three displacements along and three rotations around the three orthogonal axes). The vertical (Z axis) displacement of the distal end was set as 0 mm to simulate the sliding of the archwire through the bracket slot of the second premolar. Force was applied at different positions to simulate three types of loading (Fig. 3). For Type-1 loading, horizontal force was applied to the distal end. For Type-2 loading, force was applied between the distal key and the tube of the second molar. For Type-3 loading, a spring was added between mesial and distal loops to simulate preactivation, and then retraction force was applied as in Type 2. Stiffness of the spring was set as 15 N/mm. Preliminary experiments verified that preloading of 7.1 N, 14.2 N and 21.3 N in spring generated upward bending of the distal wire in SS DKL to preactivation angles (θ) of 5°, 10° and 15° (Fig. 2b). When the distal end was constrained in the vertical component, simulating the engagement of preactivated DKL in premolar brackets, the residual tension in the spring was 1.86 N, 3.71 N and 5.57 N, respectively. For TMA wire, preloading of 6 N, 12.4 N, 18.9 N and 25.3 N was applied accordingly to generate preactivation angles of DKL of 5°, 10°, 15° and 20°, and the residual tension after engagement was 0.97 N, 2.07 N, 3.18 N and 4.28 N, respectively. The mesial displacement of the distal end for each preactivation angle was calculated and set as the neutral position.

A retraction force from 1 to 8 N was applied in each loading condition at intervals of 1 N, and all the reaction force and resultant displacement data were collected. Force and moment reactions on the mesial end were recorded. Horizontal displacement of the distal end was recorded as extension of the archwire. The load deflection ratio (L/D) and moment/force ratio (M/F) were calculated accordingly.

## Results

**Fig. 4:**
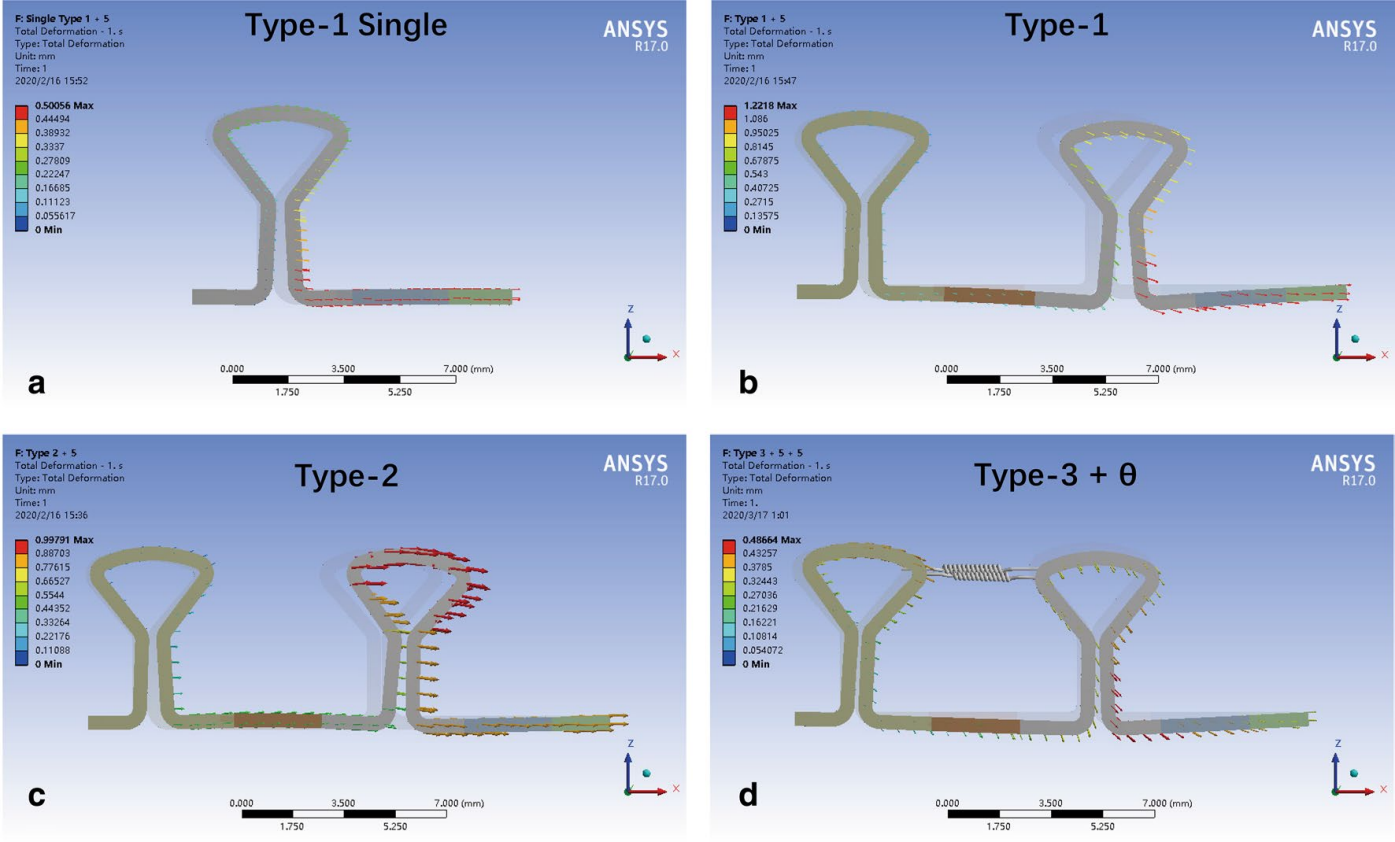
Typical proportional displacement vector of key loops after loading of 5 N distal retraction force in different types. Displacement was shown in true scale. The direction of the vectors indicated the direction of deformation, and the length of the vectors indicated the magnitude of the displacement

The deformation of SS key loops after loading of 5 N is shown in Fig. 4 and shows different patterns. The horizontal part of a single loop showed the least distal extension. In DKL, both mesial and distal loops were activated. In Type-1 loading, the distal loop opened more than the mesial loop did, and the distal loop moved occlusally. In Type-2 loading, the mesial loop opened more than the distal loop did, and the horizontal archwire was almost at the original level. Type-3 loading activated the mesial and distal loops to almost the same distance, and the distal loop occlusally shifted slightly.

**Fig. 5:**
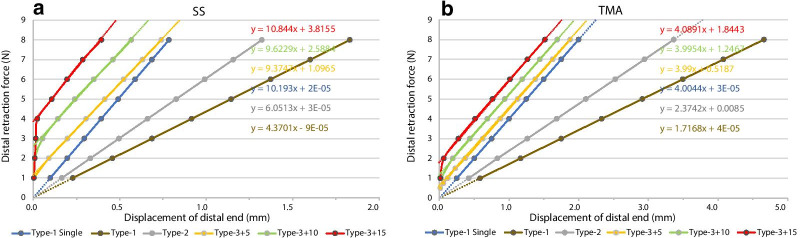
Linear fitting curve of distal retraction force against displacement for SS DKL (**a**) and TMA DKL (**b**) in different loading types. The fitted equations are displayed in the corresponding colour, and the gradient of the fitting curve indicates the load/deflection ratio of each loading condition

The displacement of the distal end under retraction force on SS loops is listed in Table 1 and Fig. 5a. For the single key loop in Type-1 loading, the L/D ratio in the horizontal direction was approximately 10.2 N/mm. For the DKL in Type-1 loading, the horizontal L/D ratio was 4.37 N/mm, approximately 43% of the single key loop. For DKL in Type-2 loading, its horizontal L/D ratio of 6.05 N/mm was higher than the horizontal L/D ratio in Type-1 loading but lower than the horizontal L/D ratio in Type-3 loading. The distal extension of DKL with preactivation angles of 5°, 10° and 15° in Type-3 loading started at retraction forces of approximately 1 N, 2 N and 4 N, respectively. The L/D ratio of DKL at a preactivation angle of 5° was 9.37 N/mm, and the ratio increased to 10.8 N/mm when the preactivation angle was up to 15°. The L/D ratio of DKL in Type-3 loading was close to the L/D ratio of a single key loop in Type-1 loading.

**Table 1:** Displacement (mm) of distal end in stainless steel single and double key loops under different loading types

Force (N)	Type-1 single	Type-1	Type-2	Type-3 +5	Type-3 +10	Type-3 +15
1	0.098	0.229	0.165	0.003	0.004	0.004
2	0.196	0.458	0.331	0.090	0.009	0.009
3	0.294	0.687	0.496	0.198	0.054	0.015
4	0.392	0.915	0.661	0.305	0.143	0.024
5	0.491	1.144	0.826	0.414	0.241	0.107
6	0.589	1.373	0.992	0.523	0.350	0.196
7	0.687	1.602	1.157	0.632	0.461	0.287
8	0.785	1.831	1.322	0.740	0.567	0.394

For TMA loops in Type-1 loading, the L/D ratio of a single key loop was 4.00 N/mm, and the L/D ratio of DKL was 1.72 N/mm. The L/D ratio of DKL in Type-2 loading was 2.37 N/mm. The distal extension of TMA DKL with preactivation angles of 5°, 10° and 15° in Type-3 loading started at a retraction force of approximately 0.5 N, 1 N and 2 N, respectively. The L/D ratio of TMA DKL under Type-3 loading was close to the L/D ratio of a single TMA key loop under Type-1 loading, remaining at approximately 4 N/mm. The results were approximately 40% of the corresponding value of SS key loops (Table 2, Fig. 5b).

**Table 2:** Displacement (mm) of distal end in TMA single and double key loops under different loading types

Force (N)	Type-1 single	Type-1	Type-2	Type-3 +5	Type-3 +10	Type-3 +15
1	0.250	0.582	0.421	0.120	0.012	0.024
2	0.499	1.165	0.832	0.371	0.191	0.063
3	0.749	1.747	1.262	0.622	0.437	0.272
4	0.999	2.330	1.683	0.873	0.688	0.512
5	1.249	2.912	2.103	1.124	0.939	0.763
6	1.498	3.495	2.524	1.374	1.190	1.013
7	1.748	4.077	2.945	1.625	1.440	1.264
8	1.998	4.660	3.365	1.876	1.691	1.515

The vertical fore at the mesial end of the SS single and double key loops was negative in Type-1 loading regardless of the force level, indicating that an extrusive force acted on the anterior teeth. In contrast, vertical force at the mesial end was positive for DKL in Type-2 loading, meaning an intrusive force on anterior teeth. The vertical force of DKL at the mesial end in Type-3 loading after engagement (retraction force = 0) was extrusive, and the magnitude increased with the preactivation angle. At a preactivation angle of 5°, the extrusive force decreased with increasing retraction force, and the vertical force became intrusive when the retraction force was above 6 N. However, the vertical force remained extrusive in Type-3 loading at preactivation angles of 10° and 15° (Table 3). For TMA loops, vertical forces at the mesial end were similar in direction to the vertical forces at the mesial end of SS loops, but the magnitudes were less when subjected to the same retraction force. In Type-3 loading of TMA DKL with a preactivation angle of 5°, the vertical force became intrusive when the retraction force was up to 4 N (Table 4).

**Table 3:** Reaction force and moment on the mesial end of stainless-steel key loops and the moment/force ratio for different loading types

	Retraction force (N)	X (N)	Z (N)	Moment (N mm)	M/F (mm)
Type-1 single	2	2.00	− 0.742	7.06	3.53
	4	4.00	− 1.483	14.12	3.53
	6	6.00	− 2.225	21.18	3.53
Type-1	2	2.00	− 0.417	7.11	3.56
	4	4.00	− 0.835	14.22	3.56
	6	6.00	− 1.252	21.33	3.56
Type-2	2	1.98	0.223	6.48	3.27
	4	3.96	0.445	12.97	3.27
	6	5.94	0.668	19.46	3.27
Type-3 +5	0	0.00	− 0.517	8.79	–
	1	0.99	− 0.308	9.54	9.62
	2	1.98	− 0.167	11.43	5.77
	4	3.96	− 0.058	18.20	4.59
	6	5.94	0.028	25.35	4.26
Type-3 +10	0	0.00	− 1.105	18.78	–
	1	0.99	− 0.895	19.51	19.69
	2	1.98	− 0.685	20.24	10.21
	4	3.96	− 0.379	23.64	5.96
	6	5.94	− 0.202	29.26	4.92
Type-3 +15	0	0.00	− 1.690	28.73	–
	1	0.99	− 1.480	29.46	29.73
	2	1.98	− 1.273	30.23	15.26
	4	3.96	− 0.862	31.83	8.03
	6	5.94	− 0.591	35.84	6.03

**Table 4:** Reaction force and moment on the mesial end of TMA key loops and the moment/force ratio for different loading types

	Retraction force (N)	X (N)	Z (N)	Moment (N mm)	M/F (mm)
Type-1 single	2	2.00	− 0.742	7.06	3.53
	4	4.00	− 1.483	14.12	3.53
	6	6.00	− 2.225	21.18	3.53
Type-1	2	2.00	− 0.417	7.11	3.56
	4	4.00	− 0.835	14.22	3.56
	6	6.00	− 1.252	21.33	3.56
Type-2	2	1.98	0.223	6.48	3.27
	4	3.96	0.445	12.97	3.27
	6	5.94	0.668	19.46	3.27
Type-3 +5	0	0.00	− 0.023	3.68	–
	1	0.99	− 0.064	5.39	5.44
	2	1.98	− 0.003	9.14	4.61
	4	3.96	0.027	16.74	4.22
	6	5.94	0.086	24.34	4.10
Type-3 +10	0	0.00	− 0.479	8.15	–
	1	0.99	− 0.291	9.24	9.32
	2	1.98	− 0.164	11.38	5.74
	4	3.96	− 0.069	18.39	4.64
	6	5.94	− 0.010	25.99	4.37
Type-3 +15	0	0.00	− 0.745	12.67	–
	1	0.99	− 0.557	13.76	13.88
	2	1.98	− 0.378	15.02	7.58
	4	3.96	− 0.167	20.05	5.06
	6	5.94	− 0.108	27.66	4.65

Moment on the mesial end in all loading types increased with the increased distal traction force (Table 3). For SS loops in Type-1 loading, the moment increased proportionally, and the M/F ratio remained at 3.53 mm. Adding a parallel key loop in DKL induced no change in the M/F ratio. The moment in Type-2 loading of DKL also increased proportionally with the distal force, and the M/F ratio increased to 3.27 mm, which was close to the M/F ratio in Type-1 loading.

The moment in Type-3 loading of DKL increased with the preactivation angle and retraction force. After simulative engagement of SS DKL in brackets without retraction, the moments on the mesial end were 8.79, 18.78 and 28.73 N·mm at preactivation angles of 5°, 10° and 15°. All M/F ratios under Type-3 loading of DKL were higher than the M/F ratios under Type-1 and Type-2 loading. As the retraction force increased, the moment at the mesial end increased, but the M/F ratio decreased inversely. The highest M/F ratios at preactivation angles of 5°, 10° and 15° were 9.62, 19.69 and 29.73 mm, respectively, under a 1 N retraction force. The corresponding M/F ratios of DKL with preactivation angles of 5°, 10° and 15° under 6 N retraction force were 4.26, 4.92 and 6.03 mm, respectively (Table 3).

For TMA loops (Table 4), the moments at mesial end in Type-1 and Type-2 loadings under equal retraction forces were almost the same, and the corresponding M/F ratios were the same as the M/F ratios of the SS loops. In Type-3 loading of DKL, the moment at the mesial end after simulative engagement was 3.68, 8.15 and 12.67 N.mm at preactivation angles of 5°, 10° and 15°. The M/F ratio of TMA DKL under the same retraction force was lower than the M/F ratio of SS DKL with an equal preactivation angle. The highest M/F ratios at preactivation angles of 5°, 10° and 15° were 5.44, 9.32 and 13.88 mm, respectively, under a 1 N retraction force. Its M/F ratio under a 6 N retraction force varied between 4.10 and 4.65 mm.

**Fig. 6:**
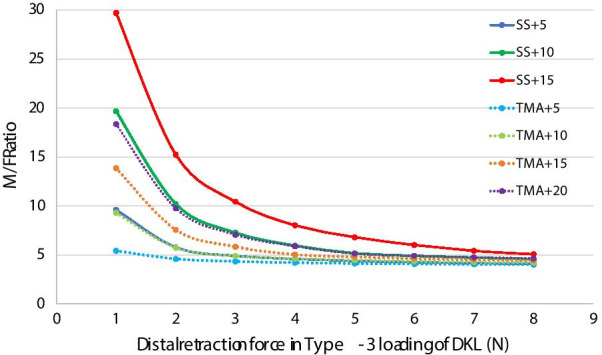
Fitting curve of the M/F ratio against the distal retraction force of SS and TMA DKL in Type-3 loading with different preactivation angles

The change in the M/F ratio against the distal retraction force is shown in Fig. 6. At the same level of retraction force, the M/F ratio increased with the preactivation angle. The M/F ratio of SS DKL was higher than the M/F ratio of TMA DKL under the same level of retraction force. Almost overlapping in the fitting curve of SS + 5 and TMA + 10 and overlapping of SS + 10 and TMA + 20 suggested their equal M/F ratio under the same retraction force.

**Fig. 7:**
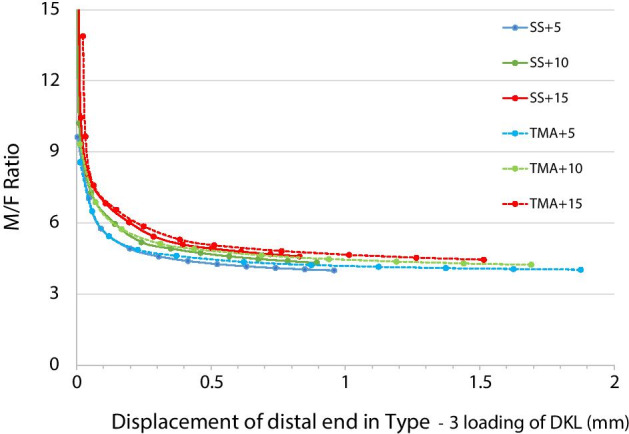
Fitting curve of the M/F ratio against the distal displacement of SS and TMA DKL in Type-3 loading with different preactivation angles

As shown in Fig. 7, the M/F ratio decreased with the extension of the distal end. The M/F ratios under all conditions were all above 4.03 mm. The M/F of DKL with a higher preactivation angle was above the M/F of DKL with lower angles. At an equal amount of distal extension, the M/F ratio of DKL increased with the preactivation angle. The fitting curves of SS and TMA DKL with equal preactivation angles were close to each other, indicating similar M/F ratios at the same distance of activation.

## Discussion·

DKL was advocated for extraction space closure, but its mechanical properties were not well defined in previous literature. In the commercial DKL products, the most ordered size of SS loops was 0.019 × 0.025 inches. Finite element analysis has been widely used in studies of orthodontic biomechanics, including appliances, wire materials, loops and force direction control [11, 12, 16, 17, 28]. For orthodontic loops, the analytical results of finite element models were consistent with experimental data in previous research [20, 29], which supported the credibility of finite element analysis in similar studies. The actual force exerted on teeth is very complicated and influenced by many factors such as the elastic property of periodontic tissue, root length, height of alveolar bone, and clearance between the archwire and bracket slot. In this study, the finite element model was simplified to be flat without teeth and curvature for comparison of different loading conditions and force levels. Stiffness of spring in Type 3 activation was critical for optimal simulation. Calculating results using different data (5, 10, 15, 30 N/mm) showed that there was few changes when the spring stiffness was over 15 N/mm. The data with spring of 15 N/mm was chosen for publication.

### Single key versus double key loop

Obviously, the double key loop had a lower L/D ratio than the single key loop. For a single key loop, a force over 10 N was needed to activate the single key loop for 1 mm in Type-1 loading. This value is quite high, and it was seldom used in clinic. SS DKL got activation of 1 mm under 4.37 N force in Type-1 loading, which was acceptable in loop mechanics for clinicians. Previous studies proved that cross-section, height of loop and wire material were three major factors affecting the L/D ratio of closing loops [11, 13]. When switched to TMA, the force required for 1 mm activation dropped by approximately 60% for single and double key loops. DKL and TMA were superior to a single key loop and SS in terms of wire rigidity.

### L/D ratio of DKL

The L/D ratio of DKL was also affected by the loading type. In Type-2 loading, the L/D ratio of SS and TMA loops was approximately 1.35 times higher than the L/D ratio of SS and TMA loops in Type-1 loading, possibly because of the change in location of the force application. When loaded at the distal key, the deformation of the mesial key was almost the same, but the distal loop showed distal tipping instead of loop opening (Fig. 4). When loaded in Type-3, the L/D ratio increased dramatically to approximately 10 and 4 N/mm for SS and TMA DKLs, respectively, which were close to the L/D ratios of single key loops. Ligation between double keys was performed with 0.0025-inch SS wire in the clinic, which kept the keys together. An elastic spring with high stiffness up to 15 N/mm was used to simulate the ligation in the model. The retraction force on the distal key was transmitted through the spring to the mesial key and finally to the mesial end. Hence, DKL behaved similarly to a single key loop in the L/D ratio.

For DKL with preactivation in Type-3 loading, distal extension was restricted when the retraction force was too low, and the retraction force induced distal tipping and occlusal movement of the distal key loop. The initial force level to start distal extension varied with the wire material and preactivation angle. The initial activating force increased with the preactivation angle, and SS DKL needed a higher force to start activation than TMA DKL. After initial activation, the DKL with preactivation showed linear extension as in other loading conditions with similar L/D ratios of a single key loop.

### Vertical force

Vertical force is important for appraising loops. Key loops in Type-1 loading exerted extrusive force at the mesial end, as reported in former research on T loops [11, 16]. In Type-2 loading of DKL, retraction force induced distal tipping of the distal loop and brought the mesial archwire and key loop above the original level, inducing intrusive force on the mesial end. These results were consistent with the photoelastic results of Dobranszki [25]. For Type-3 loading of DKL, a condition normally used for correction of deep bites in the clinic, there was extrusive force at the mesial end. The main reason was the exclusion of canines in the model. As demonstrated in Fig. 4d, ligation between key loops brought them together, inducing upward movement of mesial and distal archwire, and downward movement of the horizontal wire between loops. After engagement in the clinic, the canines would be extruded while mesial and distal teeth would be intruded due to the elasticity of the periodontic tissue. Establishment of an integrated model with bone, periodontal tissue, teeth and orthodontic appliances was necessary to fully interpret the vertical reaction of teeth to DKL.

### M/F of key loops

The M/F ratio is the key factor controlling the moving pattern of the tooth [8]. The M/F ratio is determined by the cross-section, height, loop design and preactivation [11–13]. In this study, the M/F of key loops in Type-1 loading and Type-2 loading was constant in the process of activation and did not change with the wire material. Single and double key loops showed similar M/F ratios in Type-1 loading. In Type-2 loading, the M/F ratio of DKL was close to the M/F ratio in Type-1 loading. However, the M/F ratio should be above 7 mm to attain controlled tipping of teeth and higher than 10 mm to achieve bodily movement [8]. Therefore, single and double key loops in the plain wire were not enough to achieve good control of space closure.

Methods to increase the M/F ratio of closing loops include reverse curve of Spee, V bend and gable bend [15, 17, 19]. The special preactivation method for DKL was ligation between the mesial and distal key loops, which generated upward bending of the mesial and distal archwires. Although the horizontal archwire beyond the key loops was still flat, there was a curvature in the whole archwire, which was similar to the reverse curve of Spee and the gable bend. Engagement of preactivated DKL into bracket slots induced a positive initial moment on anterior teeth. As shown in Tables 3 and 4, this initial moment increased with the preactivation angle and rigidity of the wire material. The retraction force at the distal key pulled the distal key loop backwards and induced an additional positive moment on the mesial end. However, the M/F ratio decreased with increasing retraction force. Taking SS DKL with a preactivation angle of 10° as an example, the M/F ratio was 19.69 mm when the retraction force was 1 N, but it dropped to 4.92 mm when the retraction force was 6 N. In another point of view, the M/F ratio increased with the deactivation of DKL from 6 to 1 N in Type-3 loading. The resultant movement of anterior teeth will start from control tipping and then turn into translation and root torque. When the retraction force reaches null, there will be only a positive moment at the mesial end, indicating further anterior root torque movement.

For traditional closing loops, preactivation status, including the depth of the reverse curve and angle of gable bends, tends to be weakened after several weeks in the mouth. Preactivation through ligation in DKL shows minor change because additional force is stored in the deformation of the vertical legs, and they are free from masticatory force. It is very important for clinicians to prolong the appointment intervals up to six weeks and give enough time for the expression of positive torque on anterior teeth [22]. As assumed by Dr. Kumar, the key indication for further activation was whether the angle of the canine had become normal and the arch had been levelled or not [21].

### Optimal loading condition

The force system of DKL is quite complicated, and it is not easy for clinicians to decide a suitable loading condition. As shown in Fig. 6, the M/F ratio of DKL in Type-3 loading under the same level of retraction force increased with the preactivation angle. To attain a similar M/F ratio, TMA DKL required twice the preactivation angle of SS DKL.

In orthodontic clinics, it is more convenient to observe the distal extension of the archwire than to measure the force on the ligature wire between the distal key loop and the molar tube. Normally, orthodontists activate closing loops up to 1 mm in each visit and wait enough time for the next activation. Figure 7 shows that the M/F ratio of DKL at a certain distance of activation in Type-3 loading depended mainly on the preactivation angle, while the wire material had no obvious impact. It is reasonable to select the preactivation angle according to the desired change in anterior torque based on the original status. To achieve similar torque control on anterior teeth with the same preactivation angle, TMA DKL could provide 2.5 times the longer distance of activation at equal retraction force or provide approximately 60% lower magnitude of force and moment at the same distance of activation compared with SS DKL. There could be several combinations of loop materials, preactivation angles and activation distances. These data were instructive for clinicians to make a good decision.

Traditionally, the optimal force was 3.1 N for upper anterior teeth and 2.6 N for lower anterior teeth [30, 31]. However, the latest research suggests that force with lower magnitude could be optimal for bodily orthodontic movement [32]. Activation of 0.019 × 0.025 inch SS DKL up to 1 mm in Type-3 required force that was quite heavy. Unsurprisingly, this heavy force was not realized by clinicians and called for evidence from experimental studies. If tested in actual measurements, the use of SS DKL for space closure should be reconsidered. TMA DKL of the same size required approximately 40% of the force of SS DKL for the same loading condition. Changing the DKL material into TMA is a good choice, or the cross-section and configuration should be optimized for SS DKL.

## Conclusions

The force system of DKL changed with loading type, preactivation angle and wire material. Type-2 loading of DKL showed a higher L/D ratio than Type-1 loading. Type-3 loading showed the highest M/F ratio with a similar L/D ratio of a single key loop. The M/F ratio of DKL on anterior teeth in Type-3 loading increases with the preactivation angle. The M/F ratio of DKL in Type-3 loading increased in the process of deactivation, and the M/F ratio at a certain distance of activation depended mainly on the preactivation angle instead of the wire material. DKL of TMA was recommended as a substitute for SS for a lower magnitude of force.

## Data Availability

All data generated or analysed during this study are included in this published article.

## Abbreviations

DKL: Double key loop
SS: Stainless steel
TMA: Titanium molybdenum alloy
L/D ratio: Load deflection ratio
M/F ratio: Moment/force ratio
N: Newton
